# Protocol update and baseline characteristics for the TRIal to slow the Progression of Diabetes (TRIPOD) randomized controlled trial

**DOI:** 10.1186/s13063-023-07770-7

**Published:** 2023-11-14

**Authors:** Joann Bairavi, Daphne Su-Lyn Gardner, Ester Yeoh, Kwang Wei Tham, Mihir Gandhi, Ngiap Chuan Tan, Phong Ching Lee, Robyn Su May Lim, Thofique Adamjee, Yin Bun Cheung, Yong Mong Bee, Eric Andrew Finkelstein

**Affiliations:** 1 Health Services & Systems Research, Duke-NUS Medical School, 8 College Road, Singapore, 169857 Singapore; 2 Department of Endocrinology, Singapore General Hospital, Outram Road, Singapore, 169608 Singapore; 3 Diabetes Centre, Admiralty Medical Centre, Khoo Teck Puat Hospital, Singapore, 730676 Singapore; 4 Endocrinology Services, Woodlands Health, 2 Yishun Central 2, Singapore, 768024 Singapore; 5 Centre for Quantitative Medicine, Duke-NUS Medical School, 8 College Road, Singapore, 169857 Singapore; 6 Department of Biostatistics, Singapore Clinical Research Institute, 31 Biopolis Way, Singapore, 138669 Singapore; 7 Department of Research, SingHealth Polyclinics, 167 Jalan Bukit Merah, Connection One, Tower 5, #15-10, Singapore, 150167 Singapore; 8 Health Promotion Board, 3 Second Hospital Avenue, Singapore, 168937 Singapore; 9 Department of General Medicine, Khoo Teck Puat Hospital, Singapore, 768828 Singapore

**Keywords:** Diabetes, Smartphone apps, mHealth, Behaviour change, Physical activity, Weight monitoring, Blood glucose monitoring, Medication adherence, Financial incentive

## Abstract

**Background:**

Type 2 diabetes (T2D), a major risk factor for cardiovascular disease and other adverse health conditions, is on the rise in Singapore. TRIPOD is a randomized controlled trial aimed to determine whether complementing usual care with an evidence-based diabetes management package (DMP) —comprising access to an evidence-based app, health coaching, pedometer, glucometer and weighing scale, with or without a financial rewards scheme (M-POWER rewards), can improve mean HbA_1c_ levels at months 6 and 12.

**Methods:**

The protocol was published in *Trials*, accessible via https://trialsjournal.biomedcentral.com/articles/10.1186/s13063-019-3749-x^1^. This manuscript updates the protocol with changes to the study design due to challenges with recruitment and presents baseline characteristics. Key updates include changing the arm allocation ratio from 1:1:1 (Arm 1-Usual Care: Arm 2-DMP: Arm 3-DMP+M-POWER rewards) to 10:1:10, the sample size from 339 to 269, the intervention period from two to one year, and the primary hypothesis to focus solely on differences between Usual Care and DMP+M-POWER rewards. Recruitment for the study began on 19 October 2019 and ended on 4 June 2022.

**Results:**

The average age of participants was 55.0 (SD9.7) years old and 64.2% were male. The majority of participants (76.8%) were Chinese, 4.9% Malay and 18.3% Indian and of other ethnicities. 67.0% had a monthly household income of SGD$4000 or more. The mean baseline HbA_1c_ was 8.10% (SD 0.95) and the mean body mass index was 26.8 kg/m^2^ (SD 5.3).

**Discussion:**

The final participant completed month 12 follow-up data collection on 8 June 2023. All pre-planned analyses will be conducted and final results reported.

**Trial registration:**

ClinicalTrials.govNCT03800680. Registered on 11 January 2019.

**Supplementary Information:**

The online version contains supplementary material available at 10.1186/s13063-023-07770-7.

## Background

Type 2 diabetes (T2D), a major risk factor for cardiovascular disease and other adverse health conditions, is on the rise in Singapore. TRIPOD (TRIal to slow the Progression Of Diabetes) is a randomized controlled trial with the initial objective to determine whether complementing usual care with an evidence-based diabetes management package (DMP), comprising access to an evidence-based app, health coaching, pedometer, glucometer and weighing scale, with or without financial rewards (M-POWER rewards) can improve mean glycated haemoglobin (HbA_1c_) levels at Months 6 and 12 (primary endpoint) among individuals with T2D. Analyses of mean differences between groups for changes in weight, blood pressure, proportion of participants who progress to insulin, self-reported physical activity, weight monitoring, blood glucose monitoring, medication adherence, diabetes self-management, sleep quality, work productivity, daily activity impairment and health utility index were also proposed, as was quantifying the incremental cost-effectiveness ratios (ICERs) of each intervention arm. The original protocol was published in *Trials* on 28 Nov 2019, accessible via https://trialsjournal.biomedcentral.com/articles/10.1186/s13063-019-3749-x [1].

## Methods

In the initial protocol, the plan was to recruit 339 adults with T2D using a 1:1:1 (Usual Care): diabetes management package (DMP): DMP+M-POWER rewards program (DMP + M-POWER rewards)] allocation across arms. However, due to challenges with meeting the monthly recruitment target, partly due to COVID-19, and in efforts to meet the primary study objectives given the overall budget, several changes were made to the protocol. Major changes, which were approved by the National University of Singapore (NUS)-Institutional Review Board (IRB) (Reference no. H-19-042), are listed below:

1. The arm allocation ratio was reduced from 1:1:1 to 10 (Usual Care):1 (DMP):10 (DMP + M-POWER rewards) to allow for a reduction in the overall sample size from 339 to 269 participants, with at least 113 participants in each of the Usual Care and DMP + M-POWER rewards arms and 36 in the DMP alone arm. This sample size allows for detecting a mean HbA_1c_ difference of 0.5% between the Usual Care and DMP + M-POWER rewards arms, with 80% power at 5% two-sided type I error rate assuming 20% attrition at 12 months. We removed a formal statistical test between DMP and the remaining arms. At the point when the change in allocation ratio was made, a total of 87 participants had been recruited — 30 participants were randomized into Arm 1, 27 participants to Arm 2 and 30 participants to Arm 3. Detailed changes to the primary analysis are described in Appendix 1.
2. Our primary hypothesis was changed to focus on the largest expected difference only, which is the difference between Usual Care and DMP+M-POWER rewards arms. DMP participants will be used for the process evaluation only.
3. All pre-planned primary and secondary hypotheses will be tested at months 6 and 12 only. Month 18 and 24 assessments were dropped.
4. To increase enrolment, the sampling frame was extended from 11 referral sites managed by Singapore Health Services (SingHealth) to country wide. As a result, the stratification factor- diabetes centre - was updated from ‘Polyclinic’ and ‘Specialist Clinic’ to ‘Primary Care Sector’ and ‘Secondary Care Sector’ (i.e. patients who are under the care of specialists based at re-structured or private hospitals) respectively.
5. To increase eligibility, two key changes were made: The window period for having an HbA_1c_ test (an inclusion criterion) was extended from 3 to 6 months, and (2) we removed the conditionally eligible criterion [i.e. need for the requirement for a doctor’s approval note for participants who responded, “Yes” to any of the questions in the Physical Activity Readiness Questionnaire (PARQ)]. We instead added, “Currently on doctor’s advice against engaging in moderate-to-vigorous physical activity (i.e. brisk walking or more intense)” and, “Currently have a condition(s) that restricts engaging in moderate-to-vigorous physical activity (i.e. brisk walking or more intense)” to the exclusion criteria to ensure that participants would be fit to participate in the study. Other changes to inclusion, exclusion and withdrawal criteria are shown in Appendix 1.
6. Venous HbA_1c_ samples obtained were run on Roche C513 machine from 18 August 2020 onwards due to a switch by our vendor’s laboratory. The Siemens DCA Vantage® Analyser Point-Of-Care-Testing (POCT) machine was used for all participants recruited from 5 August 2021 onwards. This allowed for flexibility in scheduling participants for their study visits. Szablowski [2] found that the Siemens DCA Vantage® is as accurate as clinical lab HbA_1c_ results. Both machines are standardized with the Diabetes Control and Complications Trial (DCCT) and National Glycohemoglobin Standardization Program (NGSP), respectively.
7. Due to a technical issue with the Welch Allyn Spot Vital Signs 4200B machine, the Omron HEM 7130 Blood Pressure monitor was used for participants who were recruited and attended their follow-up visits between 25 Jan 2021 and 25 Aug 2022. We plan to adjust for the measurement source in the analyses.
8. We had also encountered technical issues with the RxCap pill trackers and RxCap app. As a result, the issuing of the RxCap pill trackers was stopped and replaced with advice to use the Medisafe app® (Boston, Massachusetts, USA) [3] for tracking medication adherence.
9. The requirement for extraction of medical records was removed and replaced with questions on healthcare utilization to the baseline, 6- and 12-month questionnaires.
10. Changes to payouts for attending assessment sessions were made to reduce attrition. The payouts for attending the training session and completing the month 6 and month 12 assessments were increased from SGD$10 to SGD$30. The fairness payout for Arm 1 was reduced from $150 to $100 to ensure that those in the intervention arms would receive a higher payout. This resulted in the maximum cash compensation over 1 year to be $190 for those in Arm 1, $232 for those in Arm 2 and $678 for those in Arm 3. See Table 1 for revised payouts.

**Table 1:** Revised participant payouts

Type of payout	Condition	Payouts		
		Study Arm 1 (Usual Care)	Study Arm 2 (DMP)	Study Arm 3 (DMP + M-POWER rewards)
Attending training session	Attend the training session.	$30	$30	$30
Completing assessments	Complete assessments at months 6 and 12 within their respective window periods. This includes completing the follow-up questionnaire, providing photos of current diabetes medication (if any), and attending the follow-up assessments.	$30 per assessment	$30 per assessment	$30 per assessment
Incentives for app engagement	Successfully log data for weekly components: 1. Physical activity 2. Weight and blood glucose monitoring 3. Medication adherence In the respective apps: 1. Fitbit2. Glycoleap for mPower 3. RxCap At least once during the first Monday to Sunday (inclusive) of each month.	NA	$2 per app (Fitbit, Glycoleap, RxCap) per month Max earnable $6 per month (for a total of 3 apps)	$2 per app (Fitbit, Glycoleap, RxCap) per month Max earnable $6 per month (for a total of 3 apps)
M-POWER rewards	As per rewards scheme.	NA	NA	Max 516 M-Points over 1 year (1 M-Point = $1)
Fairness payout	Complete month 12 assessment within the window period.	$100 for entire study (for not receiving DMP, incentives for data upload, and M-POWER rewards)	$70 for entire study (for not receiving M-POWER rewards)	NA
**Maximum cash compensation/rewards over 1 year**		**$190**	**$232**	**$678**

**Fig. 1:**
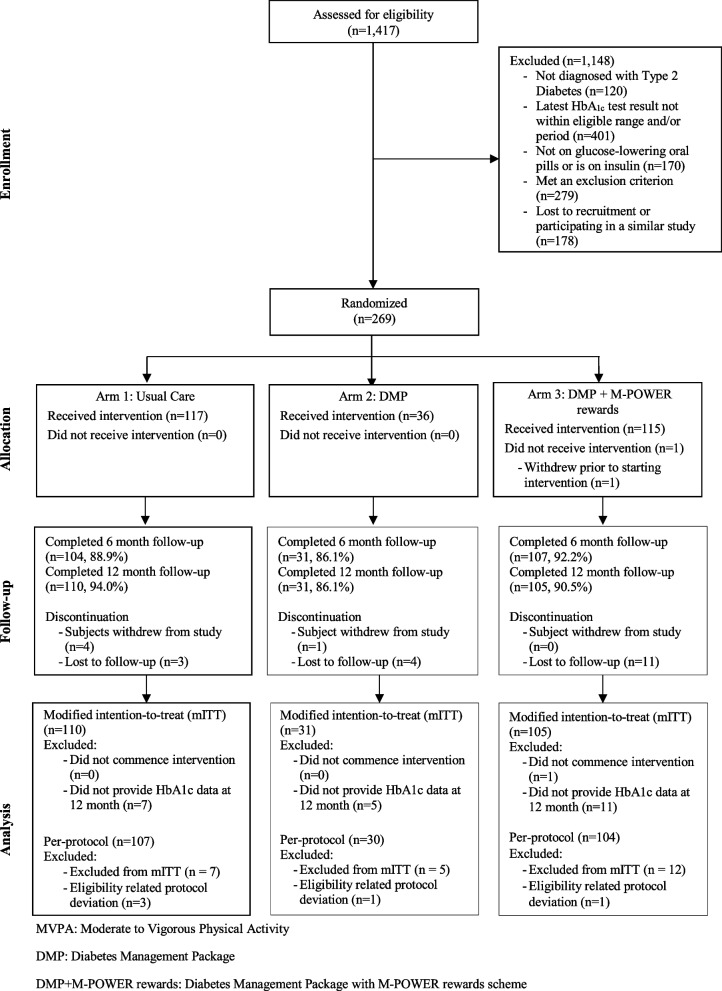
CONSORT participant flow diagram at recruitment. MVPA, Moderate to Vigorous Physical Activity; DMP, diabetes management package; DMP+M-POWER rewards, diabetes management package with M-POWER rewards scheme

This manuscript conforms to the Consolidated Standards of Reporting Trials (CONSORT) [4] statement (see Additional file 1 and Fig. 1).

## Results

One thousand four hundred seventeen individuals were assessed for eligibility. One thousand one hundred forty-eight were deemed ineligible for reasons noted in the CONSORT diagram shown in Fig. 1. On 4 June 2022, the 269th participant was recruited, with 117 randomized into Usual Care, 36 into DMP and 116 into DMP + M-POWER rewards, thus completing enrolment. The final participant completed the month 12 follow-up on 8 June 2023.

Baseline characteristics of participants are presented in Table 2. The mean age of participants was 55.0 (SD 9.7) years, and 64.2% were male. The majority of participants (76.8%) were Chinese, 4.9% were Malay and 18.3% were Indian or of other ethnicities. Most (77.6%) had post-secondary education with more than half (57.7%) working full time; 67.0% had a gross monthly household income of SGD$4000 or more with 88.2% staying in HDB/JTC flats with 4 rooms or higher. The mean BMI was 26.8kg/m^2^ (SD 5.3).

**Table 2:** Baseline characteristics

Population: modified intention-to-treat			
	Baseline (***N*** = 246)		
**Characteristics**	**Usual Care (*N*** **= 110)**	DMP* + M-POWER rewards (*N* **= 105)**	DMP* (*N* **= 31)**
Demographic characteristics			
Age (years), mean (SD)	54.7 (9.77)	54.9 (10.05)	56.1 (8.38)
Male, *n* (%)	69 (62.7)	69 (65.7)	20 (64.5)
Ethnicity, *n* (%)			
Chinese	87 (79.1)	78 (74.3)	24 (77.4)
Malay	4 (3.6)	5 (4.8)	3 (9.7)
Indian and others	19 (17.3)	22 (20.9)	4 (12.9)
Marital status, *n* (%)			
Married	80 (72.7)	76 (72.4)	23 (74.2)
Education level, *n* (%)			
Primary and below	1 (0.9)	0 (0)	0 (0)
Secondary and below	17 (15.5)	21 (20.0)	8 (25.8)
Higher than secondary	90 (81.8)	82 (78.1)	19 (61.3)
Other education levels	2 (1.8)	2 (1.9)	4 (12.9)
Employment status, *n* (%)			
Working (full-time)	66 (60.0)	57 (54.3)	19 (61.3)
Working (part-time)	13 (11.8)	19 (18.1)	3 (9.7)
Homemaker, retired or not working	31 (28.2)	29 (27.6)	9 (29.0)
Household income, *n* (%)			
< 2000 SGD	12 (10.9)	12 (11.5)	2 (6.5)
2000–3999 SGD	26 (23.6)	22 (21.2)	6 (19.4)
>= 4000 SGD	72 (65.5)	70 (67.3)	23 (74.2)
Housing type, *n* (%)			
HDB 1–3 room	14 (12.7)	11 (10.5)	4 (12.9)
HDB 4 room or higher	96 (87.3)	94 (89.6)	27 (87.1)
Disease characteristics			
HbA_1c_ (%), mean (SD)	8.22 (0.96)	7.93 (0.82)	8.25 (1.23)
Low (≤ 9.2), *n* (%)	91 (82.7)	99 (94.3)	26 (83.9)
High (> 9.2), *n* (%)	19 (17.3)	6 (5.7)	5 (16.1)
Body mass index (kg/m^2^), mean (SD)	27.0 (5.00)	26.3 (5.57)	27.2 (5.57)
Body mass index, *n* (%)			
< 18.5 kg/m^2^	1 (0.9)	4 (3.9)	1 (3.2)
18.5–22.9 kg/m^2^	28 (25.5)	25 (24.3)	4 (12.9)
23–27.4 kg/m^2^	28 (25.5)	39 (37.9)	14 (45.2)
>= 27.5 kg/m^2^	53 (48.2)	35 (34.0)	12 (38.7)
Systolic blood pressure (mmHg), mean (SD)	121.6 (14.0)	122.6 (13.4)	122.7 (10.4)
Diastolic blood pressure (mmHg), mean (SD)	75.6 (8.5)	76.7 (8.8)	77.3 (8.6)

*DMP diabetes management package

The mean HbA_1c_ was 8.10% (SD 0.95) with a majority (78.0%) having an HbA_1c_ of 7.5–11.0% at baseline; 21.1% had HbA_1c_ that was less than 7.5% and only 0.81% had HbA_1c_ that was more than 11.1%. These participants had a HbA_1c_ of 7.5–11.0% at the point of taking the screener but had different values at the baseline visit, the latter of which is used for the primary analysis.

## Discussion

Recruitment for this multi-component three-arm randomized controlled trial was challenged not only by COVID-19, but also by several competing diabetes self-management studies at the referral sites, stringent eligibility criteria and high demands of trial participants. These challenges required revisiting the study aims and making some difficult decisions in efforts to meet the primary objectives of the study given the time and budget allocated. Ultimately, we decided to scale back the primary hypothesis to test for the largest difference expected, which was the difference between usual care and the effect of the diabetes management package cum rewards program (DMP+M-POWER rewards). If this difference is not confirmed then we would not expect to see differences of the DMP without the rewards program. If the difference is confirmed then a future study may be required to tease out the independent effect of the rewards program.

The same goes for scaling back the duration of the study to not include assessments beyond 12 months which reduced the burden on trial participants who would now only have to attend three study visits at Duke-NUS Medical School instead of five study visits and to use the apps only for one year instead of two years. If differences between usual care and DMP+M-POWER rewards are identified at twelve months, then future studies may be required to test for sustained effects. If differences are not identified, then it is unlikely that they would materialize after a longer duration. Process evaluations of the DMP and DMP+M-POWER rewards arms will provide additional insight into what is driving the results and the potential for sustainability.

The changes discussed above allowed for scaling back the sample size to a manageable number and reasonable timeline, especially after adjusting our enrolment criteria and increasing our publicity efforts through multiple channels. The use of the HbA_1c_ POCT machine also allowed for increased flexibility to enrol participants who could only attend the baseline visit during non-working hours and conduct subsequent follow-up study visits.

The changes discussed above focused on increasing enrolment. Other changes were required to accommodate concerns with the intervention itself. This resulted from unexpected problems with the RxCap pill tracker and app where data was not systematically being captured and as a result not reflected on the M-POWER App. To accommodate this concern, we advised affected participants to use the Medisafe® medication adherence app® or to self-report their adherence data. Participants also encountered issues with syncing their Contour Plus One glucometer readings to the GlycoLeap app. As a result, participants were advised to manually enter their post-meal readings into the app. The M-point redemption function on the M-POWER app for Android users was also inconsistent, resulting in unclear receipts being sent for redemption. As a workaround, participants sent the receipts to the study coordinators who uploaded them on the participants’ behalf.

## Conclusion

Despite the challenges noted above, the revised protocol will allow for meeting the primary research objectives within the timeframe and budget allotted. These changes are documented in this manuscript, along with baseline characteristics of the recruited sample. Final data collection at month 12 concluded in June 2023. All pre-planned analyses will be conducted and final results reported.

### Supplementary Information

**Additional file 1:** Reporting checklist for randomised trial.

**Additional file 2:** Appendix 1.

## Data Availability

Not available.

## Abbreviations

BMI: Body mass index
CONSORT: Consolidated Standards of Reporting Trials
DCCT: Diabetes Control and Complications Trial
DMP: Diabetes management package
DSMB: Data and Safety Monitoring Board
HbA1c: Glycated haemoglobin
HDB: Housing and Development Board
ICER: Incremental cost-effectiveness ratio
JTC: Jurong Town Corporation
mHealth: Mobile health
MVPA: Moderate to Vigorous Physical Activity
NGSP: National Glycohemoglobin Standardization Program
NUS-IRB: National University of Singapore-Institutional Review Board
PARQ: Physical Activity Readiness Questionnaire
POCT: Point-Of-Care-Testing
SD: Standard deviation
T2D: Type 2 diabetes
TRIPOD: TRIal to slow the Progression Of Diabetes
