# Supplementary material for: Protocol update and baseline characteristics for the TRIal to slow the Progression of Diabetes (TRIPOD) randomized controlled trial
Source: Trials. 2023 Nov 14;24:728. doi: 10.1186/s13063-023-07770-7 (PMC10647071; doi:10.1186/s13063-023-07770-7)
Supplement: Supplementary file 2 — Additional file 2: Appendix 1. [file 13063_2023_7770_MOESM2_ESM.pdf]

Appendix 1 Changes to Inclusion, Exclusion and Withdrawal Criteria

(1) Revision to primary and secondary analysis plan

Initial Primary and Secondary Analysis Plan	*Revised Primary and Secondary Analysis Plan*
<u>Primary analysis</u><br>Mean change in HbA_{1c} level at Month 12 is the primary outcome. The primary analysis will be performed on a modified intention-to-treat population, including participants who have both baseline and Month 12 HbA_{1c} level data. A linear regression model with HbA_{1c} level at Month 12 as the dependent variable and an intercept, HbA_{1c} level at baseline (continuous variable), indicator variables for participants who received DMP alone and participants who received DMP plus M-POWER rewards, and indicator variables for stratification factors (gender and diabetes center) as independent variables will be performed. Using this model, a test for the global null hypothesis of all three arms having equal mean HbA_{1c} level at Month 12 will be performed (Null Hypothesis 1: Coefficient of DMP plus M-POWER rewards = Coefficient of DMP = 0), followed by tests for three pairwise hypotheses, comparing mean HbA_{1c} level at Month 12 in DMP alone vs. usual care (Null Hypothesis 2: Coefficient of DMP = 0), DMP plus M-POWER rewards vs. usual care (Null Hypothesis 3: Coefficient of DMP plus M-POWER rewards = 0), and DMP plus M-POWER rewards vs. DMP alone (Null Hypothesis 4: Coefficient of DMP plus M-POWER rewards = Coefficient of DMP). We will also conduct a sensitivity analysis with further adjustment for dichotomized HbA_{1c} levels at baseline (7.5–9.2% vs. 9.3–11.0%). The differences in the primary outcome between study arms will be presented along with corresponding 95% confidence intervals. Following the closed testing procedure for controlling for multiple comparisons involving three groups, only if tests of both the global null hypothesis and a pairwise null hypothesis reach statistical significance at the 0.05 level will the pairwise null hypothesis be rejected. If there are substantially different drop-out rates or different drop-out patterns among the three arms, a general linear model for repeated measures will be performed for the primary analysis. This model simultaneously models HbA_{1c} level at baseline, Month 6, and Month 12 as the dependent variables and includes	<u>Primary analysis</u><br>Mean change in HbA_{1c} level at Month 12 is the primary outcome. The primary analysis will be performed on a modified intention-to-treat population, including participants who have both baseline and Month 12 HbA_{1c} level data. A linear regression model with HbA_{1c} level at Month 12 as the dependent variable and an intercept, HbA_{1c} level at baseline (continuous variable, also a randomization stratification factor), an indicator variable for participants who received DMP plus M-POWER rewards, and indicator variables for the remaining stratification factors (gender and diabetes center) as independent variables will be performed. Using this model, a test for comparing mean HbA_{1c} level at Month 12 in DMP plus M-POWER rewards vs. usual care (Null Hypothesis: Coefficient of DMP plus M-POWER rewards= 0) will be performed. We will also conduct a supportive analysis with adjustment for dichotomized HbA_{1c} levels at baseline (7.5–9.2% vs. 9.3–11.0%) instead of its continuous format. The difference in the primary outcome between DMP plus M-POWER rewards and usual care arms will be presented along with corresponding 95% confidence interval. If there are substantially different drop-out rates or different drop-out patterns between DMP plus M-POWER rewards and usual care arms, a general linear model for repeated measures will be performed for the primary analysis. This model simultaneously models HbA_{1c} level at baseline, Month 6, and Month 12 as the dependent variables and includes interactions between indicator variables for DMP plus M-POWER rewards and Month 6 visit, and DMP plus MPOWER and Month 12 visit as independent variables. The model will also adjust for visit (indicator variable for the Month 6 visit and indicator variable for the Month 12 visit) and randomization stratification variables (gender and diabetes center). An unstructured matrix will be used to model the residual variance-covariance structure within participant. The model does not include main effect term for the intervention variable, and

interactions between indicator variables for DMP alone and Month 6 visit, DMP plus M-POWER rewards and Month 6 visit, DMP alone and Month 12 visit, and DMP plus MPOWER and Month 12 visit as independent variables. The model will also adjust for visit (indicator variable for the Month 6 visit and indicator variable for the Month 12 visit) and randomization stratification variables (gender and diabetes center). An unstructured matrix will be used to model the residual variance-covariance structure within participant. The model does not include main effect terms for the intervention variables, and thus it constrains the estimated group means of baseline HbA_{1c} levels to be identical across the three randomized groups. This model specification helps control for the variation in baseline HbA_{1c} level arising by chance among the randomized groups. Using this model, a test for the global null hypothesis of all three arms having equal mean HbA_{1c} level at Month 12 will be performed (Null Hypothesis 1: Coefficient of interaction between indicator of DMP alone and indicator of Month 12 visit = Coefficient of interaction between indicator of DMP plus M-POWER rewards and indicator of Month 12 visit = 0), followed by tests for three pairwise hypotheses, comparing mean HbA_{1c} level at Month 12 in DMP alone vs. usual care (Null Hypothesis 2: Coefficient of interaction between indicator of DMP alone and indicator of Month 12 visit = 0), DMP plus M-POWER rewards vs. usual care (Null Hypothesis 3: Coefficient of interaction between indicator of DMP plus M-POWER rewards and indicator of Month 12 visit = 0), and DMP plus M-POWER rewards vs. DMP alone (Null Hypothesis 4: Coefficient of interaction between indicator of DMP alone and indicator of Month 12 visit = Coefficient of interaction between indicator of DMP plus M-POWER rewards and indicator of Month 12 visit). If the missing data patterns do not necessitate using a general linear model for repeated measures as a primary analysis, this analysis will be conducted as a sensitivity analysis.<br><u>Secondary effectiveness analyses</u><br>**Quantitative outcomes**<br>Secondary quantitative outcomes (weight, blood pressure, GPAQ total physical activity score, weight monitoring frequency, blood	thus it constrains the estimated group means of baseline HbA_{1c} levels to be identical across the two randomized groups. This model specification helps control for the variation in baseline HbA_{1c} level arising by chance among the randomized groups. Using this model, a test for DMP plus M-POWER rewards vs. usual care (Null Hypothesis: Coefficient of interaction between indicator of DMP plus M-POWER rewards and indicator of Month 12 visit = 0) will be performed. If the missing data patterns do not necessitate using a general linear model for repeated measures as a primary analysis, this analysis will be conducted as a supportive analysis.<br><u>Secondary effectiveness analyses</u><br>**Quantitative outcomes**<br>Secondary quantitative outcomes (weight, blood pressure, GPAQ total physical activity score, weight monitoring frequency, blood glucose monitoring frequency, diabetes medication adherence frequency, DSMQ sum score, global PSQI score, percent overall work impairment and percent activity impairment due to diabetes and related health problems using a modified WPAI:SHP, health utility index using 5-level EQ-5D) will be analyzed using a similar strategy as applied to the primary outcome with. A linear regression model will be used to model the secondary quantitative outcome as the dependent variable and an intercept, the outcome at baseline (quantitative variable), indicator variables for participants who received DMP plus M-POWER, indicator variables for stratification factors (gender, diabetes center), and HbA_{1c} level at baseline (quantitative variable) as independent variables. Additional analyses will also be performed to evaluate the intervention effects that account for insulin progression/medication changes and other potential effect modifiers, mediators, covariates, and program engagement metrics.<br>**Binary outcomes**<br>Secondary binary outcomes (e.g., proportion of participants who had insulin treatment initiated by their diabetes care physician) will be analysed using a generalized linear model with a logit link function and binomial distribution (log-binomial regression model). The model will include the following as independent variables: an intercept, indicator variables for participants

glucose monitoring frequency, diabetes medication adherence frequency, DSMQ sum score, global PSQI score, percent overall work impairment and percent activity impairment due to diabetes and related health problems using a modified WPAI:SHP, health utility index using 5-level EQ-5D) will be analyzed using a similar strategy as applied to the primary outcome. A linear regression model will be used to model the secondary quantitative outcome as the dependent variable and an intercept, the outcome at baseline (quantitative variable), indicator variables for participants who received DMP alone and participants who received DMP plus M-POWER, and indicator variables for stratification factors (gender, diabetes center, and dichotomized HbA_{1c} level at baseline) as independent variables. Additional analyses will also be performed to evaluate the intervention effects that account for insulin progression/medication changes and other potential effect modifiers, mediators, covariates, and program engagement metrics.<br>**Binary outcomes**<br>Secondary binary outcomes (e.g., proportion of participants who had insulin treatment initiated by their diabetes care physician) will be analysed using a generalized linear model with a log link function and binomial distribution (log-binomial regression model). The model will include the following as independent variables: an intercept, indicator variables for participants who received DMP alone and participants who received DMP plus M-POWER, and indicator variables for stratification factors (gender, diabetes center, and dichotomized HbA_{1c} level at baseline).	who received DMP plus M-POWER, indicator variables for stratification factors (gender, diabetes center), and HbA_{1c} level at baseline (quantitative variable).

(2) Revisions to inclusion criteria

Additional revision was made as follows:

(i) The words, “with no plans to relocate during the study period” was added to exclude individuals who may not be able to participate in this study for 1 year and attend all 3 study visits.

Initial Inclusion criteria	*Revised Inclusion Criteria*
1. Have been diagnosed with T2D with sub-optimal diabetes control as defined by an HbA_{1c} level between 7.5 and 11.0% (inclusive) at the most recent test taken within the past 3 calendar months<br>2. Are not on insulin<br>3. Are on at least one oral glucose-lowering drug<br>4. Are aged 21 to 70 at last birthday<br>5. Are Singapore citizens or permanent residents<br>6. Are able to read, write, and communicate in English<br>7. Own a personal smartphone and are comfortable<br>with using apps.	1. Diagnosed with T2D with suboptimal diabetes control as defined by a HbA_{1c} level of between 7.5% and 11.0% (inclusive) at their most recent test taken within the past six calendar months. This HbA_{1c} inclusion criterion will be based on the patients’ self-reported HbA_{1c} levels and test dates.<br>2. Not on insulin.<br>3. On at least one oral glucose-lowering drug.<br>4. Aged between 21 and 70 (inclusive) at last birthday.<br>5. Singapore Citizen or Permanent Resident with no plans to relocate during the study period.<br>6. Able to read, write, and communicate in English.<br>7. Own a personal smartphone and be able to use it.

(3) Revisions to exclusion criteria

Additional revisions were made as follows:

(i) ”Have a history of chronic kidney disease” and, “Have undergone dialysis for treatment of kidney failure” were merged to, ”Diagnosed with chronic kidney disease [stage 3B with estimated Glomerular Filtration Rate (eGFR) <45mL/min] or undergoing dialysis for end-stage kidney failure”.

(ii) “Have a history of cardiovascular disease” was revised to, “Diagnosed with heart attack (i.e., acute myocardial infarction) within the past one year” and “Diagnosed with heart failure (i.e., congestive heart failure)”.

(iii) “Have a history of stroke” was revised to, “Diagnosed with stroke or transient ischemic attacks”

(iv) ”Have a history of blood diseases” was revised to, “Diagnosed with severe anaemia (Haemoglobin <10g/dL)”,”Diagnosed with sickle-cell disease”, and ,”Diagnosed with Thalassemia major”.

(v) “Have a history of chronic liver disease” was revised to, “Diagnosed with liver cirrhosis”.

(vi) “Have undergone chemotherapy, radiation therapy, or immunotherapy for cancer treatment in the past 5 years” was revised to, “Diagnosed with cancer that required treatment in the past five years”.

(vii) “Have undergone blood transfusion in the past 3 months” was revised to, “Undergone whole blood or red blood cell transfusion within the past three months”.

(viii) “Are taking systemic corticosteroids” was revised to, “Taking systemic corticosteroids (including Traditional Chinese or Malay medicine)”.

(ix) “Have had any major surgery in the past year”, and, “Are unable to walk up 10 stair steps (individual steps, not floors) without stopping/difficulty” were removed as the two criteria on inability to engage in moderate-to-vigorous activity were deemed adequate in excluding those who were unable to walk regularly.

(x) A criterion, “Undergone lower limb amputation (including toe amputation)” was added to exclude those with a history of lower limb amputation(s).

Initial Exclusion criteria	*Revised Exclusion Criteria*
1. Are pregnant or lactating<br>2. Have a history of chronic kidney disease<br>3. Have undergone dialysis for treatment of kidney failure<br>4. Have a history of cardiovascular disease<br>5. Have a history of stroke<br>6. Have a history of blood diseases<br>7. Have a history of chronic liver disease<br>8. Have undergone chemotherapy, radiation therapy, or immunotherapy for cancer treatment in the past 5 years<br>9. Have undergone blood transfusion in the past 3 months<br>10. Are taking systemic corticosteroids<br>11. Have a history of bariatric surgery or extensive bowel resection<br>12. Have had any major surgery in the past year<br>13. Are unable to walk up 10 stair steps (individual steps, not floors) without stopping/difficulty.	1. Pregnant or lactating.<br>2. Diagnosed with chronic kidney disease (stage 3B with eGFR <45mL/min) or undergoing dialysis for end-stage kidney failure.<br>3. Diagnosed with liver cirrhosis.<br>4. Diagnosed with cancer that required treatment in the past five years.<br>5. Diagnosed with heart attack (i.e., acute myocardial infarction) within the past one year.<br>6. Diagnosed with heart failure (i.e., congestive heart failure)<br>7. Diagnosed with stroke or transient ischemic attacks.<br>8. Undergone whole blood or red blood cell transfusion within the past three months.<br>9. Diagnosed with severe anaemia (Haemoglobin <10g/dL)<br>10. Diagnosed with sickle-cell disease<br>11. Diagnosed with Thalassemia major<br>12. Undergone bariatric surgery or extensive bowel resection.<br>13. Undergone lower limb amputation (including toe amputation).<br>14. Taking systemic corticosteroids (including Traditional Chinese or Malay medicine).<br>15. Currently on doctor’s advice against engaging in moderate-to-vigorous physical activity (i.e., brisk walking or more intense).<br>16. Currently have a condition(s) that restricts engaging in moderate-to-vigorous physical activity (i.e., brisk walking or more intense).

(4) Revision of withdrawal criteria

Following the change to the exclusion criteria, “Diagnosed with heart attack (i.e., acute myocardial infarction) within the past one year”, “Diagnosed with heart failure (i.e., congestive heart failure)” and, “Diagnosed with stroke or transient ischemic attacks”, participants who are diagnosed with heart attack (i.e., acute myocardial infarction), heart failure (i.e., congestive heart failure), or stroke / transient ischemic attacks during their study period, we added to our withdrawal criteria that study team member(s) may consider stopping participants’ participation.

As this trial includes effectiveness aims, we will not withdraw participants on grounds other than participants’ volition and/or safety since outcomes that are unlikely to be affected by conditions that develop or treatments that are received during the study period can still be analyzed in accordance with effectiveness approaches. From an effectiveness approach, we are executing a pragmatic trial that will be describing observed effects under real-world conditions. E.g. Participants who are diagnosed with cancer and/ or need to undergo cancer treatment during the course of their participation will not be withdrawn from the study unless they voluntarily choose to do so or their doctor deems their continued participation unsafe.

Initial Withdrawal criteria	*Revised Withdrawal criteria*
Participants are free to withdraw from the study at any<br>time by informing the study team or the investigators of<br>their decision to withdraw. Data that has been collected<br>until the time of their withdrawal will be stored and analyzed.<br>Participants may be discontinued from the study<br>due to one or more of the following reasons:<br>1. They become pregnant.<br>2. Upon voluntarily informing their doctor that they<br>are participating in this study, their doctor decides<br>that continuing participation could be harmful and<br>informs us in the process.<br>3. They fail to follow the instructions of the study<br>team or investigators.<br>Participants who develop any of the exclusion criteria<br>2–13 during the course of the study will not be withdrawn from the study unless they choose to withdraw<br>voluntarily. There are no concomitant care or other interventions that will be prohibited during the<br>study.	Participation in this study is entirely voluntary and participants can withdraw at any time. Participants will be instructed to inform the study team members and/or the Principal Investigator on their decision to withdraw. They will be informed that the data that has been collected until the time of their withdrawal will be kept and analysed to enable a complete and comprehensive evaluation of the study.<br><br>Participants will be informed that their doctor, the PI, and/or study team members may consider stopping their participation at any time due to one or more of the following reasons:<br>• They become pregnant<br>• They are diagnosed with heart attack (i.e., acute myocardial infarction), heart failure (i.e., congestive heart failure), or stroke / transient ischemic attacks within their study period<br>• Their attending doctor decides that continuing participation could be harmful due to the participant developing medical conditions or receiving treatment that could affect his/her safety when engaging in the study intervention, especially for physical activity.<br>• Failure to follow the instructions of the study team or PI<br>• The study is cancelled

	If recruited participants develop conditions or undergo procedures that are listed as exclusion criteria during the course of their participation, they will not be withdrawn unless (1) they voluntarily choose to do so, (2) their doctor deems their continued participation unsafe, or (3) they become pregnant.
